# Más allá del volumen corpuscular medio: el papel del volumen de los neutrófilos y de los monocitos en el diagnóstico del déficit de B12 en pacientes con anemia

**DOI:** 10.1515/almed-2025-0163

**Published:** 2025-11-13

**Authors:** Alba Leis-Sestayo, Álvaro Piedra-Aguilera, Laura Jiménez-Añón, Jennifer Rodríguez-Domínguez, Carme García-Martín, Alicia Martínez-Iribarren, Cristian Morales-Indiano

**Affiliations:** Servicio de Análisis Clínicos y Bioquímica, Hospital Universitario Germans Trias i Pujol, Badalona, Barcelona, España

**Keywords:** vitamina B12, datos sobre población celular, volumen corpuscular medio

Estimado Editor,

El déficit de vitamina B12 (B12) es la principal causa de anemia megaloblástica [1] pudiendo deberse a una ingesta dietética insuficiente, a trastornos gastrointestinales que ocasionen malabsorción o a defectos congénitos que afectan al transporte o a la absorción de la cobalamina. En términos clínicos, el déficit de vitamina B12 se caracteriza por un amplio espectro de manifestaciones hematológicas y neurológicas, por lo que un diagnóstico temprano y preciso resulta crucial a la hora de prevenir daños neurológicos irreversibles.

Las características hematológicas más frecuentes del déficit de vitamina B12 incluyen: anemia, macrocitosis, presencia de neutrófilos hipersegmentados y cambios megaloblásticos en sangre periférica y médula ósea; todos ellos reflejo de una síntesis de ADN alterada [1], 2]. Sin embargo, la presencia de neutrófilos hipersegmentados o de un volumen corpuscular medio elevado (VCM) en el frotis de sangre periférica no son indicadores sensibles del déficit de B12 [1], 3], 4].

El analizador hematológico Beckman Coulter DxH900 proporciona los denominados datos sobre la población celular (CPD) mediante la tecnología VCS, que combina la medición del volumen (V, a través de la impedancia), la conductividad (C, a través de radiofrecuencia) y la dispersión de la luz (S, a través de la dispersión láser multiangular), permitiendo así obtener datos cuantitativos sobre las características morfológicas de las diferentes poblaciones leucocitarias.

En el presente estudio, evaluamos y comparamos el rendimiento diagnóstico del VCM y los CPD a la hora de identificar el déficit de B12 en pacientes de atención primaria en España.

Para tal fin, realizamos un estudio retrospectivo entre enero de 2022 y marzo de 2024, en el que se clasificó a los pacientes en tres grupos: grupo control (B12>300 pg/mL), grupo 2 (pacientes con déficit de B12, B12<187 pg/mL), y grupo 3 (pacientes con déficit severo de B12, B12<100 pg/mL). El grupo control se dividió a su vez en dos subgrupos: 1a y 1b, con el objeto de equilibrar la distribución entre sexos.

La totalidad de los pacientes presentaban anemia (definida como niveles de hemoglobina <12 g/dL para las mujeres y <13 g/dL para los hombres), así como niveles de ferritina de entre 30 y 150 ng/mL, un recuento leucocitario <10 × 10^9^/L, y niveles de alanina aminotransferasa <50 UI/L. Los criterios de exclusión fueron una tasa de filtración glomerular estimada <40 mL/min/1,73 m^2^, niveles de proteína C-reactiva >10 mg/L, diagnóstico de talasemia o embarazo. Se incluyó a pacientes de entre 18 y 80 años, excluyendo del grupo de control a aquellos pacientes con déficit de folato. El presente estudio fue revisado y aprobado por el Comité de Ética (código del CEIC PI-24-220).

El análisis estadístico se llevó a cabo con el programa MedCalc v19.6 (Ostend, Bélgica). Se evaluó la utilidad diagnóstica del VCM y de los CPD mediante la prueba *t* de Student y a través del análisis de curvas ROC.

Se recogieron los datos de VCM y los CPD de volumen, conductividad y dispersión láser. Se observaron diferencias estadísticamente significativas entre el grupo de control y los grupos con déficit de B12 en el volumen medio de los neutrófilos (MN-V-Ne) y en el volumen medio de los monocitos (MN-V-Mo) (Tabla 1). Al comparar el grupo 1a y el grupo 2, tanto el MN-V-Ne como el MN-V-Mo mostraron mejor precisión diagnóstica que el VCM (AUC=0,709 y 0,706, respectivamente).

**Tabla 1:** Análisis comparativo de los datos de las poblaciones celulares entre el grupo control y los pacientes con déficit de B12: (A) B12<187 pg/mL y (B) B12<100 pg/mL.

(A)					
	Grupo 1a (media±SD)	Grupo 2 (media±SD)	p-Value	AUC (Intervalo de confianza del 95 %)	p
Hombre/mujer (total)	36/40 (76)	36/40 (76)			
Edad, años	64,6	65,6			
Vitamina B12, pg/mL	467,8 ± 145,4	145,1 ± 31,0			
Hemoglobina, g/dL	11'9 ± 0,8	11,7 ± 0,8			
VCM, fL	88,7 ± 5,1	92,8 ± 9,7	0,015	0,608 (0,525–0,686)	0,019
MN-V-Ne	148,1 ± 6,2	152,8 ± 7,6	<0,001	0,709 (0,630–0,780)	<0,001
MN-V-Mo	174,6 ± 7,8	179,8 ± 8,6	<0,001	0,706 (0,627–0,777)	<0,001
MN-V-Ne x MN-V-Mo	258,8 ± 20,0	275,2 ± 24,8	<0,001	0,732 (0,654–0,801)	<0,001

(B)					
	Grupo 1b (media±SD)	Grupo 3 (media±SD)	p-Value	AUC (Intervalo de confianza del 95 %)	p
Hombre/mujer (total)	19/40 (59)	10/21 (31)			
Edad, años	64,1	68,6			
Vitamina B12, pg/mL	465,3 ± 144,1	86,7 ± 5,6			
Hemoglobina, g/dL	11,8 ± 0,7	11,3 ± 1,0			
VCM, fL	89,2 ± 4,7	105,4 ± 12,3	<0,001	0,887 (0,803–0,944)	<0,001
MN-V-Ne	148,0 ± 6,5	161,0 ± 10,8	<0,001	0,864 (0,775–0,927)	<0,001
MN-V-Mo	174,3 ± 8,3	184,9 ± 11,8	<0,001	0,776 (0,676–0,857)	<0,001
VCM x MN-V-Ne	132,0 ± 8,5	170,7 ± 29,8	<0,001	0,921 (0,845–0,967)	<0,001

SD, desviación estándar; AUC, área bajo la curva; MN-V-Ne, volumen medio de neutrófilos; MN-V-Mo, volumen medio de monocitos; p, valor p. MN-V-Ne × MN-V-Mo y VCM × MN-V-Mo representan el resultado de los dos parámetros dividido entre 100.

La combinación de MN-V-Ne × MN-V-Mo mostró un rendimiento diagnóstico aún superior (AUC=0,732; sensibilidad=60,5 % y especificidad=80,2 %, punto de corte>269,4) (Tabla 1A, Figura 1A). Al comparar el grupo 1b con el grupo 3, tanto el VCM como el MN-V-Ne mostraron un sólido rendimiento diagnóstico, siendo su combinación (VCM × MN-V-Ne) la que mostró mayor área bajo la curva (AUC=0,921; sensibilidad=83,9 %, especificidad=89,8 %, punto de corte>141,5) (Tabla 1B, Figura 1B).

**Figura 1:**
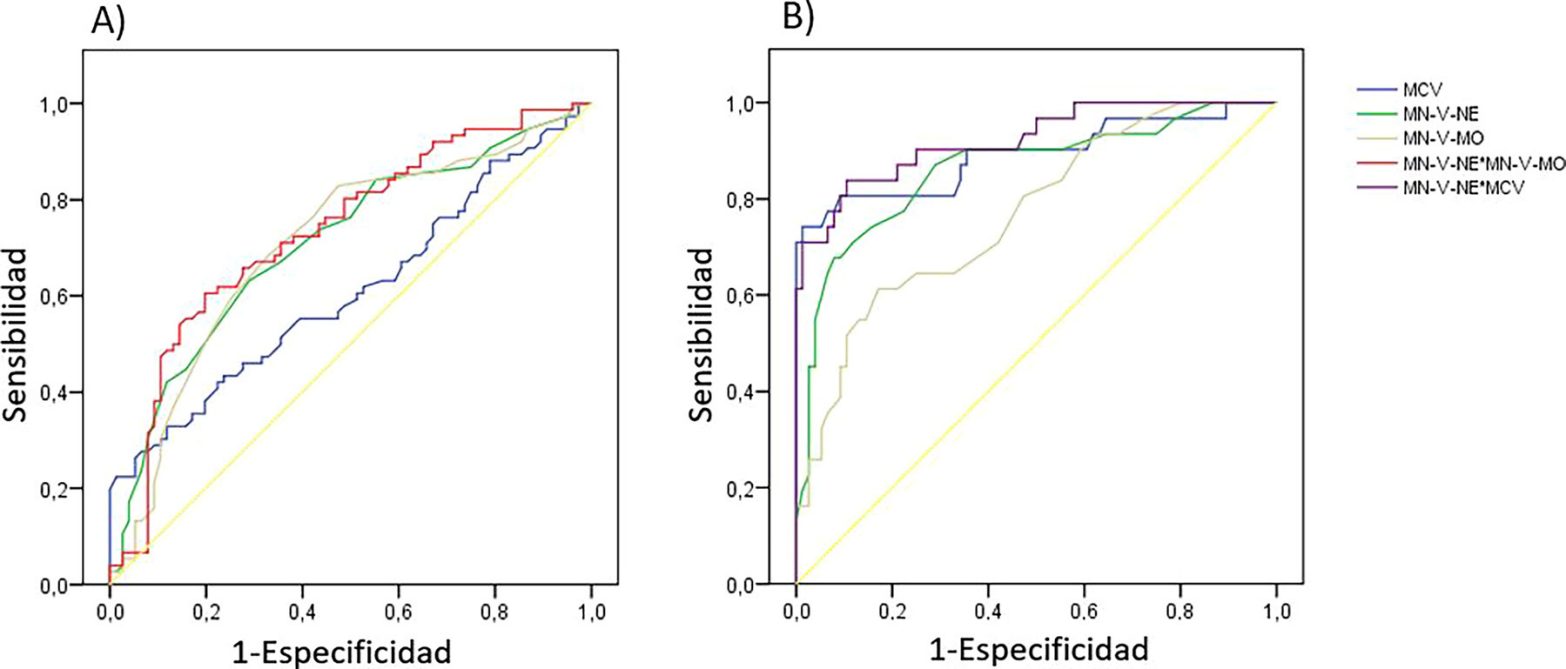
Curvas ROC en las que se compara a los pacientes con déficit de B12 con los controles. (A) B12<187 pg/mL, (B) B12<100 pg/mL.

Saha y col. [5], así como Simón-López y col. [6], que emplearon un analizador más antiguo, el Beckman Coulter LH 750, ya observaron una asociación entre el volumen de neutrófilos y monocitos y niveles bajos de B12 o de folato. Con respecto a nuestro estudio, observamos diferencias significativas en los resultados de MN-V-Ne y MN-V-Mo entre el grupo control y los grupos con déficit de B12, obtenidos con el analizador más moderno DxH 900. Así mismo, demostramos que el déficit severo de B12 influye notablemente en el rendimiento diagnóstico del VCM y de los CPD.

En el contexto del déficit de B12, el VCM muestra una sensibilidad diagnóstica limitada, especialmente en los casos leves. Por el contrario, en los casos de déficit grave de B12 (B12<100 pg/mL), el rendimiento diagnóstico del VCM mejora significativamente, lo que lo convierte en una valiosa herramienta para la detección del déficit de B12.

Los resultados de este estudio demuestran que el rendimiento diagnóstico del volumen corpuscular medio (VCM) se puede mejorar incorporando datos sobre poblaciones celulares (CPD). Así, la integración de intervalos de referencia estimados [7] similares a los empleados en el VCM, o el establecimiento de puntos de corte diagnósticos para estos parámetros de CPD en los sistemas informáticos, podrían facilitar la detección del déficit de vitamina B12.

Una limitación del presente estudio es el hecho de no haber evaluado el impacto del déficit concomitante de folato y B12, ya que el déficit de folato también se asocia a cambios megaloblásticos en las células hematopoyéticas, y la concurrencia de ambos déficits podría mejorar aún más su rendimiento diagnóstico.

En conclusión, los CPD se postulan como una herramienta útil en la detección del déficit de B12 en pacientes con anemia. Estos marcadores complementan al VCM, proporcionando apoyo diagnóstico adicional sin coste alguno, dado que estos resultados se generan automáticamente en los hemogramas rutinarios. Esta estrategia podría permitir diagnósticos más precisos y precoces, especialmente en los casos complejos de déficit de B12, o ante alteraciones de VCM causadas por otros tipos de anemia, tratamientos o patologías subyacentes.

## References

[1] Green R (2017). Vitamin B12 deficiency from the perspective of a practicing hematologist. Blood.

[2] Devalia V, Hamilton MS, Molloy AM (2014). Guidelines for the diagnosis and treatment of cobalamin and folate disorders. Br J Haematol.

[3] Snow CF (1999). Laboratory diagnosis of vitamin B12 and folate deficiency: a guide for the primary care physician. Arch Intern Med.

[4] Aslinia F, Mazza JJ, Yale SH (2006). Megaloblastic anemia and other causes of macrocytosis. Clin Med Res.

[5] Saha D, Anandarama D, Karthick RG, Vishnu M, Rao P, Suresh P (2020). The role of volume, conductivity, scatter changes of neutrophils and monocytes in diagnosis of megaloblastic anemia. Ann Pathol Lab Med.

[6] Simón-López R, Egorova M, Tsvetaeva N, Sukhacheva E, Kolenkin S, Achildieva T (2012). A novel approach for the screening of megaloblastic anemia. J US China Med Sci.

[7] Haeckel R (2021). Indirect approaches to estimate reference intervals. J Lab Med.

